# 2-Aza­niumylcarba-*closo*-dodeca­borate ethanol monosolvate

**DOI:** 10.1107/S1600536811006222

**Published:** 2011-02-26

**Authors:** Alexander Himmelspach, Guido J. Reiss, Maik Finze

**Affiliations:** a Institut für Anorganische Chemie und Strukturchemie, Lehrstuhl II: Material- und Strukturforschung, Heinrich-Heine-Universität Düsseldorf, Universitätsstrasse 1, D-40225 Düsseldorf, Germany

## Abstract

Two formula units of the title compound, 2-H_3_N-*closo*-1-CB_11_H_11_·CH_3_CH_2_OH or CH_14_B_11_N·C_2_H_5_OH, form a ring motif of *R*_4_^2^(8) type in the solid state that surrounds a crystallographic center of symmetry. The ring motif is a result of N—H⋯O hydrogen bonds. In contrast to many structures of {*closo*-1-CB_11_} clusters, the assignment of the position of the cluster C atom in the structure of the title compound is unambigious. The relatively long B—N bond length [1.5396 (10) Å] documents the absence of any B—N π-inter­action in the title compound although this was observed for a related 2-amino­carba-*closo*-dodeca­borate.

## Related literature

For a general overview on monocarba-*closo*-dodeca­borates, see: Körbe *et al.* (2006 ▶). For the synthesis and properties of 2-amino- and 2-azaniumyl­carba-*closo*-dodeca­boron clusters, see: Finze (2009 ▶). For structures and properties of related {*closo*-1-CB_11_} clusters with NH_2_ and NH_3_ groups, see: Jelínek *et al.* (1986 ▶); Finze (2007 ▶); Finze *et al.* (2007 ▶); Finze & Sprenger (2010 ▶). For hydrogen-bond motifs, see: Etter (1990 ▶).
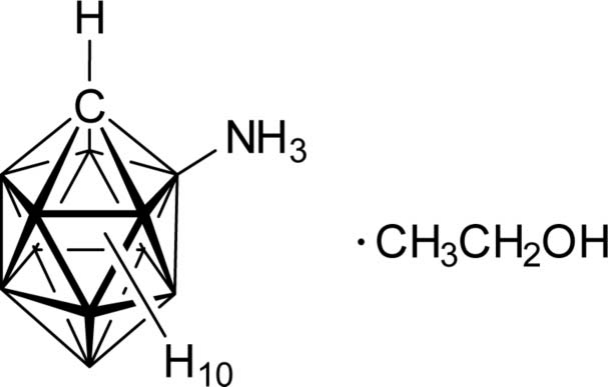


## Experimental

### 

#### Crystal data

CH_14_B_11_N·C_2_H_6_O
*M* *_r_* = 205.11
Monoclinic, 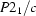

*a* = 9.5753 (2) Å
*b* = 9.2549 (2) Å
*c* = 13.9095 (5) Å
β = 97.519 (3)°
*V* = 1222.04 (6) Å^3^
*Z* = 4
Mo *K*α radiation
μ = 0.06 mm^−1^
*T* = 120 K
0.28 × 0.26 × 0.20 mm

#### Data collection

Oxford Diffraction Xcalibur Eos diffractometer
Absorption correction: multi-scan (*CrysAlis PRO*; Oxford Diffraction, 2009 ▶) *T*_min_ = 0.729, *T*_max_ = 1.000
61096 measured reflections
3561 independent reflections
3199 reflections with *I* > 2σ(*I*)
*R*_int_ = 0.030
3 standard reflections every 60 min  intensity decay: none

#### Refinement

*R*[*F* ^2^ > 2σ(*F* ^2^)] = 0.029
*wR*(*F* ^2^) = 0.069
*S* = 1.02
3561 reflections
208 parameters
H atoms treated by a mixture of independent and constrained refinement
Δρ_max_ = 0.31 e Å^−3^
Δρ_min_ = −0.22 e Å^−3^

### 

Data collection: *CrysAlis PRO* (Oxford Diffraction, 2009 ▶); cell refinement: *CrysAlis PRO*; data reduction: *CrysAlis PRO*; program(s) used to solve structure: *SHELXS97* (Sheldrick, 2008 ▶); program(s) used to refine structure: *SHELXL97* (Sheldrick, 2008 ▶); molecular graphics: *DIAMOND* (Brandenburg, 2011) ▶; software used to prepare material for publication: *SHELXL97*.

## Supplementary Material

Crystal structure: contains datablocks I, global. DOI: 10.1107/S1600536811006222/br2161sup1.cif

Structure factors: contains datablocks I. DOI: 10.1107/S1600536811006222/br2161Isup2.hkl

Additional supplementary materials:  crystallographic information; 3D view; checkCIF report

## Figures and Tables

**Table 1:** Hydrogen-bond geometry (Å, °)

*D*—H⋯*A*	*D*—H	H⋯*A*	*D*⋯*A*	*D*—H⋯*A*
N1—H1*B*⋯O1	0.900 (12)	2.006 (12)	2.8937 (9)	168.5 (10)
N1—H1*A*⋯O1^i^	0.898 (12)	2.065 (12)	2.9446 (9)	166.1 (10)

Symmetry code: (i) 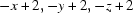.

**Fig. 1.**
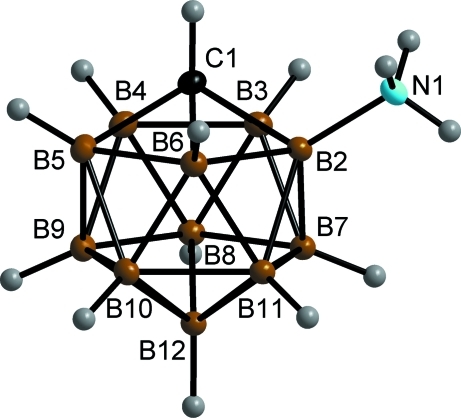
In the carborane 2-H3N-closo-1-CB11H11 the hydrogen atoms are drawn with an arbitrary radius and the displacement ellipsoids are shown at the 50% probability level.

**Fig. 2.**
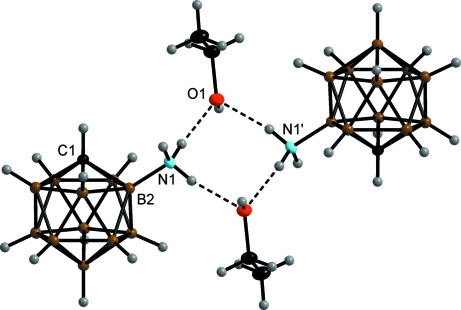
Hydrogen-bonded motif formed by 2-H3N-closo-1-CB11H11.CH3CH2OH (Symmetry code: ' = -x + 2, -y + 2, -z + 2; hydrogen atoms are drawn with an arbitrary radius and the displacement ellipsoids are shown at the 50% probability level).

## supplementary crystallographic information

### Comment

Icosahedral monocarba-*closo*-dodecaborates with functional groups, for example amino or ammonio that are bonded to the cluster atoms are building blocks for a broad range of applications (Körbe *et al.*, 2006). The properties and the reactivity of the amino groups depend on (i) the further substituents of the {*closo*-1-CB_11_} cluster and (ii) the type of the cluster atom, either carbon or boron that it is bonded to. The influence of the substituents is evident from a comparison of the properties of [1-H_2_N-*closo*-1-CB_11_*X*_11_]^-^  with X equal to either H or F. The non-fluorinated anion is indefinitely stable in concentrated aqueous bases and acids whereas the fluorinated anion decomposes in acidic aqueous solutions and undergoes substituent exchange reactions in basic aqueous solutions (Finze *et al.*, 2007). Furthermore, [1-H_2_N-*closo*-1-CB_11_F_11_]^-^ reacts with strong non-nucleophilic bases under aprotic conditions to result in a cluster rearrangement that so far was observed for highly fluorinated aminocarba-*closo*-dodecaborates only (Finze, 2007). The difference in the properties of amino groups that are either bonded to the cluster carbon atom or to one of the boron atoms is demonstrated by a comparison of the p*K*_a_value of 1-H_3_N-*closo*-CB_11_H_11_ 6.0 (Jelínek *et al.*, 1986) and of 2-H_3_N-*closo*-CB_11_H_11_ >10.5 (Finze, 2009).

The inner salt 2-H_3_N-*closo*-1-CB_11_H_11_ crystallizes as ethanol solvate in the monoclinic space group *P*2_1_/*c* with one complete molecule and one ethanol molecule in the asymmetric unit. The cluster carbon atom is unambiguously assigned as evident from comparative refinements (Figure 1). Furthermore, the experimental inner cluster carbon-boron and boron-boron bond lengths are in excellent agreement to values derived from DFT calculations at the B3LYP/6–311++G(d,p) and at the MP2/6–311++G(d,p) level of theory, which were reported earlier (Finze, 2009). In this earlier contribution it was concluded on the basis of experimental inner-cluster *d*(C—B) and *d*(B—B) of the related {*closo*-1-CB_11_} species [1-Ph-2-H_2_N-*closo*-1-CB_11_H_10_]^-^ and 1-Ph-2-Me_3_N-*closo*-1-CB_11_H_10_ in conjunction with theoretical bond lengths of [2-H_2_N-*closo*-1-CB_11_H_11_]^-^ and 2-H_3_N-*closo*-1-CB_11_H_11_ that there is a small amount of B—N π-interaction for the amino derivatives whereas for the ammonio derivatives this π-interaction was not observed. The *d*(B2—N1) of 1.5396 (10) Å in the structure of the title compound supports these previous results. As a consequence of this weakened B2—N1 bond the inner-cluster C1—B2 bond is strengthened as documented by *d*(C1—B2) of 1.6872 (11) Å.

The title compound 2-H_3_N-*closo*-1-CB_11_H_11_.CH_3_CH_2_OH forms dimers in the solid state (Table 1, Figure 2). The hydrogen-bonded ring consists of two ammonio derivatives and two ethanol molecules and has to be described as *R*^2^_4_(8) (Etter, 1990). A very similar hydrogen-bonded system was previously found for the related ammonio substituted carborane 1-H_3_N-2-F-*closo*-1-CB_11_H_10_ in the structure of its acetone solvate (Finze & Sprenger, 2010).

### Experimental

2-H_3_N-*closo*-1-CB_11_H_11_ was synthesized according to a published procedure that also includes the spectroscopic data (Finze, 2009). The title compound was dissolved in a minimum amount of diethyl ether, ethanol, and chloroform (20:1:1). The clear colorless solution was stored at 3 °C in a refrigerator resulting in colorless crystals within two days.

A single crystal suitable for structure determination was harvested under a dry nitrogen atmosphere and was directly transferred into the cooling stream of an Oxford-Xcalibur diffractometer equipped with an EOS-CCD detector.

### Refinement

All hydrogen atoms were located from difference Fourier synthesis. The H atoms of the 2-aminocarba-*closo*-dodecaborane and the H atom of the hydroxy group of the ethanol molecule were refined without any restraints. For the H atoms of the CH_2_ and CH_3_ group a riding model was employed and for each group a common *U*_iso_ value was refined.

### Crystal data

**Table:**

CH_14_B_11_N·C_2_H_6_O	*F*(000) = 432
*M**_r_* = 205.11	*D*_x_ = 1.115 Mg m^−^^3^
Monoclinic, *P*2_1_/*c*	Mo *K*α radiation, λ = 0.71073 Å
Hall symbol: -P 2ybc	Cell parameters from 48838 reflections
*a* = 9.5753 (2) Å	θ = 3.0–35.4°
*b* = 9.2549 (2) Å	µ = 0.06 mm^−^^1^
*c* = 13.9095 (5) Å	*T* = 120 K
β = 97.519 (3)°	Block, colourless
*V* = 1222.04 (6) Å^3^	0.28 × 0.26 × 0.20 mm
*Z* = 4	

### Data collection

**Table:**

Oxford Diffraction Xcalibur Eos diffractometer	3199 reflections with *I* > 2σ(*I*)
Radiation source: fine-focus sealed tube	*R*_int_ = 0.030
graphite	θ_max_ = 30.0°, θ_min_ = 4.3°
ω scans	*h* = −13→13
Absorption correction: multi-scan (*CrysAlis PRO*; Oxford Diffraction, 2009)	*k* = −13→13
*T*_min_ = 0.729, *T*_max_ = 1.000	*l* = −19→19
61096 measured reflections	3 standard reflections every 60 min
3561 independent reflections	intensity decay: none

### Refinement

**Table:**

Refinement on *F*^2^	Primary atom site location: structure-invariant direct methods
Least-squares matrix: full	Secondary atom site location: difference Fourier map
*R*[*F*^2^ > 2σ(*F*^2^)] = 0.029	Hydrogen site location: difference Fourier map
*wR*(*F*^2^) = 0.069	H atoms treated by a mixture of independent and constrained refinement
*S* = 1.02	*w* = 1/[σ^2^(*F*_o_^2^) + 0.6*P*] where *P* = (*F*_o_^2^ + 2*F*_c_^2^)/3
3561 reflections	(Δ/σ)_max_ < 0.001
208 parameters	Δρ_max_ = 0.31 e Å^−^^3^
0 restraints	Δρ_min_ = −0.22 e Å^−^^3^

### Special details

**Table:**

Experimental. *CrysAlis PRO*, Oxford Diffraction Ltd., Version 1.171.33.52 (release 06–11-2009). Numerical absorption correction based on Gaussian integration over a multifaceted crystal model..
Geometry. All e.s.d.'s (except the e.s.d. in the dihedral angle between two l.s. planes) are estimated using the full covariance matrix. The cell e.s.d.'s are taken into account individually in the estimation of e.s.d.'s in distances, angles and torsion angles; correlations between e.s.d.'s in cell parameters are only used when they are defined by crystal symmetry. An approximate (isotropic) treatment of cell e.s.d.'s is used for estimating e.s.d.'s involving l.s. planes.
Refinement. Refinement of *F*^2^ against ALL reflections. The weighted *R*-factor *wR* and goodness of fit *S* are based on *F*^2^, conventional *R*-factors *R* are based on *F*, with *F* set to zero for negative *F*^2^. The threshold expression of *F*^2^ > σ(*F*^2^) is used only for calculating *R*-factors(gt) *etc*. and is not relevant to the choice of reflections for refinement. *R*-factors based on *F*^2^ are statistically about twice as large as those based on *F*, and *R*- factors based on ALL data will be even larger.

### Fractional atomic coordinates and isotropic or equivalent isotropic displacement parameters (Å^2^)

**Table:**

	*x*	*y*	*z*	*U*_iso_*/*U*_eq_	
C1	0.64079 (8)	0.84830 (8)	0.78113 (5)	0.01344 (14)	
H1	0.5827 (11)	0.9122 (12)	0.8122 (7)	0.018 (3)*	
B2	0.80596 (8)	0.81753 (9)	0.83584 (6)	0.01164 (14)	
N1	0.85434 (7)	0.89268 (8)	0.93327 (5)	0.01414 (13)	
H1A	0.9385 (13)	0.8604 (13)	0.9609 (8)	0.027 (3)*	
H1B	0.8607 (12)	0.9893 (14)	0.9280 (8)	0.026 (3)*	
H1C	0.7976 (12)	0.8764 (13)	0.9748 (8)	0.024 (3)*	
B3	0.67585 (9)	0.68137 (9)	0.83322 (6)	0.01329 (15)	
H3	0.6339 (11)	0.6570 (11)	0.8994 (7)	0.018 (3)*	
B4	0.56348 (9)	0.70103 (10)	0.72203 (6)	0.01463 (16)	
H4	0.4514 (11)	0.6832 (12)	0.7204 (8)	0.022 (3)*	
B5	0.62540 (9)	0.85015 (9)	0.65759 (6)	0.01452 (16)	
H5	0.5509 (11)	0.9214 (12)	0.6171 (7)	0.019 (3)*	
B6	0.77708 (9)	0.92320 (9)	0.72901 (6)	0.01289 (15)	
H6	0.7950 (11)	1.0393 (11)	0.7353 (7)	0.019 (3)*	
B7	0.84826 (8)	0.63822 (9)	0.80794 (6)	0.01176 (14)	
H7	0.9196 (11)	0.5751 (12)	0.8583 (7)	0.019 (3)*	
B8	0.69640 (9)	0.56516 (9)	0.73553 (6)	0.01280 (15)	
H8	0.6694 (11)	0.4511 (12)	0.7382 (7)	0.020 (3)*	
B9	0.66519 (9)	0.66986 (9)	0.62665 (6)	0.01348 (15)	
H9	0.6180 (11)	0.6216 (12)	0.5578 (7)	0.019 (3)*	
B10	0.79711 (9)	0.80728 (9)	0.63111 (6)	0.01288 (15)	
H10	0.8360 (11)	0.8478 (11)	0.5659 (7)	0.018 (3)*	
B11	0.91068 (8)	0.78779 (9)	0.74285 (6)	0.01167 (15)	
H11	1.0217 (11)	0.8179 (11)	0.7508 (8)	0.019 (3)*	
B12	0.84085 (8)	0.63102 (9)	0.67959 (6)	0.01164 (14)	
H12	0.9092 (11)	0.5566 (11)	0.6440 (7)	0.017 (2)*	
O1	0.88753 (6)	1.20322 (7)	0.94495 (5)	0.01863 (12)	
H1O	0.9206 (15)	1.2323 (16)	0.9014 (10)	0.045 (4)*	
C2	0.78862 (9)	1.30879 (9)	0.97377 (6)	0.02120 (17)	
H2A	0.7249	1.3401	0.9176	0.035 (2)*	
H2B	0.8390	1.3926	1.0021	0.035 (2)*	
C3	0.70730 (10)	1.23985 (10)	1.04632 (7)	0.02545 (19)	
H3A	0.7713	1.2070	1.1008	0.043 (2)*	
H3B	0.6550	1.1592	1.0170	0.043 (2)*	
H3C	0.6435	1.3092	1.0677	0.043 (2)*	

### Atomic displacement parameters (Å^2^)

**Table:**

	*U*^11^	*U*^22^	*U*^33^	*U*^12^	*U*^13^	*U*^23^
C1	0.0119 (3)	0.0125 (3)	0.0161 (3)	0.0009 (3)	0.0025 (3)	−0.0011 (3)
B2	0.0120 (3)	0.0121 (3)	0.0109 (3)	−0.0006 (3)	0.0020 (3)	−0.0010 (3)
N1	0.0162 (3)	0.0148 (3)	0.0116 (3)	−0.0014 (2)	0.0026 (2)	−0.0018 (2)
B3	0.0134 (3)	0.0129 (4)	0.0140 (3)	−0.0008 (3)	0.0036 (3)	−0.0005 (3)
B4	0.0116 (3)	0.0141 (4)	0.0181 (4)	−0.0007 (3)	0.0016 (3)	−0.0019 (3)
B5	0.0141 (4)	0.0135 (4)	0.0153 (4)	0.0017 (3)	−0.0007 (3)	−0.0001 (3)
B6	0.0140 (3)	0.0113 (3)	0.0134 (3)	−0.0001 (3)	0.0021 (3)	0.0003 (3)
B7	0.0123 (3)	0.0114 (3)	0.0118 (3)	0.0001 (3)	0.0022 (3)	0.0007 (3)
B8	0.0130 (3)	0.0117 (3)	0.0139 (3)	−0.0011 (3)	0.0026 (3)	−0.0005 (3)
B9	0.0139 (3)	0.0128 (4)	0.0133 (3)	0.0002 (3)	0.0002 (3)	−0.0010 (3)
B10	0.0151 (4)	0.0119 (3)	0.0116 (3)	0.0003 (3)	0.0016 (3)	0.0005 (3)
B11	0.0118 (3)	0.0117 (3)	0.0117 (3)	−0.0005 (3)	0.0024 (3)	0.0000 (3)
B12	0.0124 (3)	0.0111 (3)	0.0115 (3)	0.0004 (3)	0.0022 (3)	0.0000 (3)
O1	0.0193 (3)	0.0187 (3)	0.0190 (3)	0.0011 (2)	0.0069 (2)	0.0008 (2)
C2	0.0232 (4)	0.0159 (4)	0.0251 (4)	0.0040 (3)	0.0055 (3)	0.0024 (3)
C3	0.0242 (4)	0.0254 (4)	0.0286 (4)	0.0045 (3)	0.0108 (3)	0.0023 (4)

### Geometric parameters (Å, °)

**Table:**

C1—B2	1.6872 (11)	B5—B6	1.7821 (12)
C1—B3	1.7207 (12)	B5—H5	1.075 (10)
C1—B4	1.7094 (12)	B6—B10	1.7636 (12)
C1—B5	1.7055 (12)	B6—B11	1.7832 (12)
C1—B6	1.7196 (11)	B6—H6	1.090 (11)
C1—H1	0.953 (10)	B7—B12	1.7787 (11)
B2—N1	1.5396 (10)	B7—B8	1.7896 (12)
B2—B11	1.7589 (11)	B7—B11	1.7985 (12)
B2—B7	1.7634 (12)	B7—H7	1.083 (10)
B2—B3	1.7691 (12)	B8—B12	1.7809 (12)
B2—B6	1.7702 (12)	B8—B9	1.7898 (12)
N1—H1A	0.898 (12)	B8—H8	1.088 (11)
N1—H1B	0.900 (12)	B9—B12	1.7822 (12)
N1—H1C	0.857 (12)	B9—B10	1.7878 (12)
B3—B8	1.7641 (12)	B9—H9	1.099 (10)
B3—B4	1.7742 (12)	B10—B11	1.7856 (12)
B3—B7	1.7777 (12)	B10—B12	1.7937 (12)
B3—H3	1.075 (10)	B10—H10	1.091 (10)
B4—B9	1.7692 (12)	B11—B12	1.7811 (12)
B4—B8	1.7816 (12)	B11—H11	1.091 (10)
B4—B5	1.7885 (13)	B12—H12	1.110 (10)
B4—H4	1.083 (11)	O1—C2	1.4532 (10)
B5—B10	1.7761 (12)	O1—H1O	0.768 (15)
B5—B9	1.7770 (12)	C2—C3	1.4958 (12)
			
B2—C1—B5	114.11 (6)	B11—B6—H6	125.5 (6)
B2—C1—B4	113.81 (6)	B5—B6—H6	121.8 (5)
B5—C1—B4	63.16 (5)	B2—B7—B3	59.94 (5)
B2—C1—B6	62.60 (5)	B2—B7—B12	106.02 (6)
B5—C1—B6	62.71 (5)	B3—B7—B12	106.91 (6)
B4—C1—B6	115.05 (6)	B2—B7—B8	106.64 (6)
B2—C1—B3	62.53 (5)	B3—B7—B8	59.27 (5)
B5—C1—B3	114.82 (6)	B12—B7—B8	59.88 (5)
B4—C1—B3	62.29 (5)	B2—B7—B11	59.17 (4)
B6—C1—B3	114.99 (6)	B3—B7—B11	107.76 (6)
B2—C1—H1	117.9 (6)	B12—B7—B11	59.72 (5)
B5—C1—H1	118.2 (6)	B8—B7—B11	107.85 (6)
B4—C1—H1	118.1 (6)	B2—B7—H7	120.6 (6)
B6—C1—H1	117.5 (6)	B3—B7—H7	121.1 (5)
B3—C1—H1	117.3 (6)	B12—B7—H7	124.6 (5)
N1—B2—C1	118.49 (6)	B8—B7—H7	123.9 (6)
N1—B2—B11	125.67 (6)	B11—B7—H7	121.2 (6)
C1—B2—B11	106.60 (6)	B3—B8—B12	107.41 (6)
N1—B2—B7	124.58 (6)	B3—B8—B4	60.05 (5)
C1—B2—B7	106.76 (6)	B12—B8—B4	107.30 (6)
B11—B2—B7	61.41 (5)	B3—B8—B9	107.38 (6)
N1—B2—B3	118.02 (6)	B12—B8—B9	59.88 (5)
C1—B2—B3	59.66 (5)	B4—B8—B9	59.39 (5)
B11—B2—B3	109.94 (6)	B3—B8—B7	60.03 (5)
B7—B2—B3	60.43 (5)	B12—B8—B7	59.76 (5)
N1—B2—B6	119.03 (6)	B4—B8—B7	108.02 (6)
C1—B2—B6	59.59 (5)	B9—B8—B7	107.76 (6)
B11—B2—B6	60.70 (5)	B3—B8—H8	120.9 (5)
B7—B2—B6	110.51 (6)	B12—B8—H8	123.2 (5)
B3—B2—B6	110.12 (6)	B4—B8—H8	121.2 (6)
B2—N1—H1A	112.1 (7)	B9—B8—H8	122.7 (6)
B2—N1—H1B	113.1 (7)	B7—B8—H8	121.8 (6)
H1A—N1—H1B	107.4 (10)	B4—B9—B5	60.58 (5)
B2—N1—H1C	111.7 (8)	B4—B9—B12	107.78 (6)
H1A—N1—H1C	105.5 (10)	B5—B9—B12	108.06 (6)
H1B—N1—H1C	106.6 (10)	B4—B9—B10	108.28 (6)
C1—B3—B8	104.97 (6)	B5—B9—B10	59.77 (5)
C1—B3—B2	57.81 (5)	B12—B9—B10	60.32 (5)
B8—B3—B2	107.51 (6)	B4—B9—B8	60.07 (5)
C1—B3—B4	58.54 (5)	B5—B9—B8	108.71 (6)
B8—B3—B4	60.47 (5)	B12—B9—B8	59.81 (5)
B2—B3—B4	106.86 (6)	B10—B9—B8	108.47 (6)
C1—B3—B7	104.68 (6)	B4—B9—H9	121.0 (5)
B8—B3—B7	60.70 (5)	B5—B9—H9	121.0 (6)
B2—B3—B7	59.63 (5)	B12—B9—H9	122.5 (5)
B4—B3—B7	108.88 (6)	B10—B9—H9	121.8 (5)
C1—B3—H3	118.3 (6)	B8—B9—H9	121.4 (6)
B8—B3—H3	128.6 (6)	B6—B10—B5	60.46 (5)
B2—B3—H3	118.2 (6)	B6—B10—B11	60.32 (5)
B4—B3—H3	121.0 (5)	B5—B10—B11	108.48 (6)
B7—B3—H3	125.7 (5)	B6—B10—B9	108.17 (6)
C1—B4—B9	104.15 (6)	B5—B10—B9	59.81 (5)
C1—B4—B3	59.17 (5)	B11—B10—B9	107.78 (6)
B9—B4—B3	107.84 (6)	B6—B10—B12	107.86 (6)
C1—B4—B8	104.69 (6)	B5—B10—B12	107.59 (6)
B9—B4—B8	60.54 (5)	B11—B10—B12	59.68 (5)
B3—B4—B8	59.49 (5)	B9—B10—B12	59.69 (5)
C1—B4—B5	58.31 (5)	B6—B10—H10	121.1 (6)
B9—B4—B5	59.93 (5)	B5—B10—H10	121.5 (5)
B3—B4—B5	108.24 (6)	B11—B10—H10	121.5 (5)
B8—B4—B5	108.57 (6)	B9—B10—H10	122.2 (6)
C1—B4—H4	119.8 (6)	B12—B10—H10	122.4 (6)
B9—B4—H4	126.9 (6)	B2—B11—B12	106.11 (6)
B3—B4—H4	119.3 (6)	B2—B11—B6	59.96 (5)
B8—B4—H4	125.9 (6)	B12—B11—B6	107.55 (6)
B5—B4—H4	120.0 (6)	B2—B11—B10	106.46 (6)
C1—B5—B10	104.26 (6)	B12—B11—B10	60.38 (5)
C1—B5—B9	103.98 (6)	B6—B11—B10	59.23 (5)
B10—B5—B9	60.42 (5)	B2—B11—B7	59.42 (5)
C1—B5—B6	59.03 (5)	B12—B11—B7	59.59 (4)
B10—B5—B6	59.42 (5)	B6—B11—B7	108.32 (6)
B9—B5—B6	107.84 (6)	B10—B11—B7	108.14 (6)
C1—B5—B4	58.52 (5)	B2—B11—H11	121.8 (6)
B10—B5—B4	107.94 (6)	B12—B11—H11	123.8 (6)
B9—B5—B4	59.50 (5)	B6—B11—H11	120.5 (6)
B6—B5—B4	108.23 (6)	B10—B11—H11	122.5 (6)
C1—B5—H5	119.9 (6)	B7—B11—H11	122.0 (6)
B10—B5—H5	126.7 (6)	B7—B12—B8	60.37 (5)
B9—B5—H5	126.7 (6)	B7—B12—B11	60.69 (5)
B6—B5—H5	119.7 (6)	B8—B12—B11	109.01 (6)
B4—B5—H5	119.7 (6)	B7—B12—B9	108.58 (6)
C1—B6—B10	104.21 (6)	B8—B12—B9	60.31 (5)
C1—B6—B2	57.80 (4)	B11—B12—B9	108.23 (6)
B10—B6—B2	106.93 (6)	B7—B12—B10	108.66 (6)
C1—B6—B11	104.15 (6)	B8—B12—B10	108.60 (6)
B10—B6—B11	60.45 (5)	B11—B12—B10	59.93 (5)
B2—B6—B11	59.34 (5)	B9—B12—B10	59.99 (5)
C1—B6—B5	58.26 (5)	B7—B12—H12	121.5 (5)
B10—B6—B5	60.12 (5)	B8—B12—H12	121.3 (5)
B2—B6—B5	106.55 (6)	B11—B12—H12	121.4 (5)
B11—B6—B5	108.32 (6)	B9—B12—H12	121.4 (5)
C1—B6—H6	118.9 (5)	B10—B12—H12	121.3 (5)
B10—B6—H6	129.1 (5)	C2—O1—H1O	109.4 (11)
B2—B6—H6	118.1 (5)	O1—C2—C3	108.36 (7)

### Hydrogen-bond geometry (Å, °)

**Table:**

*D*—H···*A*	*D*—H	H···*A*	*D*···*A*	*D*—H···*A*
N1—H1B···O1	0.900 (12)	2.006 (12)	2.8937 (9)	168.5 (10)
N1—H1A···O1^i^	0.898 (12)	2.065 (12)	2.9446 (9)	166.1 (10)

Symmetry codes: (i) −*x*+2, −*y*+2, −*z*+2.
